# How can we sustainably assess the shelf life of EVOO? A systematic review on analytical strategies and food waste reduction

**DOI:** 10.3389/fnut.2025.1722145

**Published:** 2026-01-08

**Authors:** Monica Di Maria, Diego Planeta, Francesca Venturini, Federica Torregrossa, Pasquale Crupi

**Affiliations:** 1 Department of Agricultural, Food and Forestry Sciences, University of Palermo, Palermo, Italy; 2 School of Engineering, ZHAW Zurich University of Applied Sciences, Winterthur, Switzerland; 3 TOELT LLC, Research and Development, Dubendorf, Switzerland

**Keywords:** food waste reduction, non-invasive methods, primary shelf-life, quality, secondary shelf-life, sustainable analysis, virgin olive oil

## Abstract

Nowadays, olive oil quality is assessed through a combination of physicochemical parameters and sensory evaluation performed by trained tasting panels. The International Olive Council (IOC) and Codex Alimentarius define the legal limits and reference values for these parameters. The current analytical methods used to characterize extra virgin olive oil (EVOO), are based on destructive chemical techniques that are time-consuming, require long sample preparation (highly skilled operators), resource-intensive and involve the use of toxic solvents, with marked environmental impact and costs representing an obstacle in the green transition. Additionally, many of them do not allow for real-time analysis or analysis in line with industrial processes. Furthermore, none of the currently established methods adequately address the qualitative deterioration of virgin olive during storage. Thus, quality evaluation should include not only regulatory criteria but also parameters related to human health, the formation of degradation products, especially with respect to storage conditions. In addition, attention should be focused particularly on the concept of secondary shelf-life, with the aim of reducing food waste of olive oil while characteristics still remain unaltered. In light of this, it is necessary to explore alternative analytical strategies that are rapid, non-destructive, and sustainable, capable of guaranteeing the quality and safety of EVOO, reducing food waste, and respecting the principles of environmental sustainability. This review aims to critically analyze the latest analytical methodologies applied to determine the shelf life of EVOO, with a particular focus on their potential contribution to reducing waste and aligning with the objectives of Agenda 2030.

## 1 Introduction

**Figure 1:**
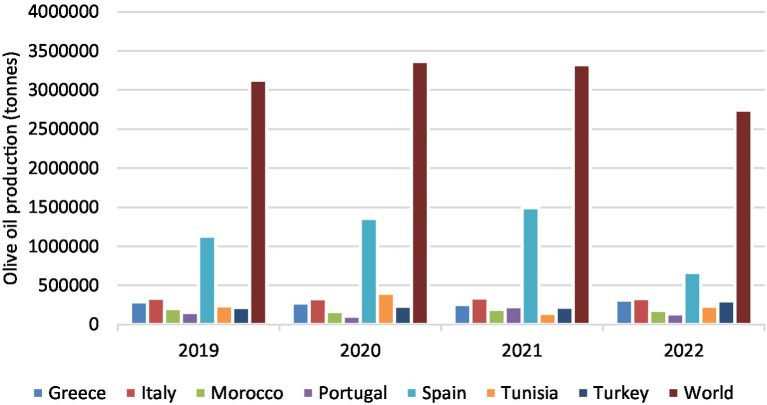
Olive oil production and main producing countries (4).

Olive oil (OO) represents a characteristic component of the Mediterranean diet and is distinguished by its sensory attributes, nutritional profile, and recognized health-promoting effects (1). OO is known to provide important health benefits thanks to its specific composition of unsaturated fatty acids, polyphenols, vitamin E, carotenoids, sterols, etc. Nowadays, there is growing consumer interest in incorporating dietary antioxidants for their potential short- and long-term health benefits, including the prevention and mitigation of gastrointestinal diseases such as colon cancer (2). In 2020, the global area cultivated with olive trees was estimated at 12.8 million hectares, predominantly allocated to oil production and largely concentrated within the Mediterranean basin (3). According to the most recent Food and Agriculture Organization of the United Nations – Statistic Division (FAOSTAT) estimations, world olive oil production in 2020 was approximately 2.7 million tons, with marked interannual variability observed over the previous 4 years (Figure 1). Nearly 90% of the total output is accounted for by eight countries: Spain (40.2%), Tunisia (11.1%), Italy (9.8%), Greece (9.1%), Turkey (7.1%), Morocco (4.9%), Syria (4.1%), and Portugal (3.2%) (4). Among these, Spain represents the leading producer and exporter of olive oil (5), exerting a dominant influence on global production trends (Figure 1).

Moreover, the world has been trying to steer its choices toward sustainability. The olive oil industry is increasingly aligning its practices with the Sustainable Development Goals (SDGs), with a strong emphasis on reducing food loss and waste (FLW) for achieving important environmental, social, and economic improvements. For instance, accurate determination of the shelf life of extra virgin olive oil (EVOO) not only has economic implications, but is also closely linked to global sustainability goals, in particular Goal 9 (industry, innovation and infrastructure) and Goal 12 (responsible consumption and production) of the 2030 Agenda. As pointed out by recent research, the olive oil sector is called upon to reduce its environmental impact throughout the entire production chain by promoting more efficient and sustainable practices (6–8). In response, countries worldwide are seeking effective strategies for the prevention and reduction of FLW, in line with Target 12.3 of the United Nations SDGs (9). These actions are key priorities on the political agendas of both national governments and international organizations (10–12); they explicitly addressed within the framework of the SDGs, a global initiative launched by the United Nations in 2015 as part of the 2030 Agenda for Sustainable Development, which established a 15-year roadmap for achieving 17 goals designed to promote prosperity while safeguarding the planet. Each SDG is defined by specific, measurable targets; in particular, Target 12.3 calls for halving per capita global food waste at the retail and consumer levels and reducing food loss along production and supply chains, including post-harvest stages, by 2030. This objective is embedded within SDG 12, which focuses on sustainable consumption and production patterns, thereby requiring the consideration of the entire agri-food chain, from primary production to end consumers, in the design of FLW prevention strategies. Furthermore, the reduction of FLW is a central pillar of the Farm to Fork Strategy (launched in 2019), which aims to enhance sustainability throughout the food chain while explicitly targeting FLW reduction (13). Consumer behavior plays a critical role, as food choice and household practice directly affect domestic waste generation (14). As highlighted by Rohm et al. (15), one considerable driver of consumer-level waste is the tendency to discard food items approaching or slightly exceeding their expiration date, even when they are still safe and suitable for consumption. In this sense, EVOO shelf life (SL) remains a crucial issue, influenced by numerous chemical, physical, and environmental factors. Although the concept of primary shelf life (PSL, the stability period before the package is opened) is widely regulated and monitored, secondary shelf life (SSL, the period during which the product maintains acceptable quality characteristics once opened) remains largely unexplored and rarely valued (16, 17). In a context where there is growing attention to sustainability and reducing food waste, assessing the SSL of EVOO takes on strategic significance.

Additionally, the application of sustainable, non-destructive analytical methods, which are characterized by low environmental impact, non-invasiveness, and minimal solvent consumption, aligns SL assessment with green chemistry principles and promotes a circular approach to oil production.

This review aims first and foremost to provide updates on the current situation regarding the SL of OO sector. Secondly, it highlights the importance of SSL and at the same time how it could prevent waste. Thirdly, this review presents the progress made in the technologies applied to determine SL, with a focus on non-destructive technologies presenting them as an alternative to destructive analysis in terms of effectiveness, speed, environmental impact, and potential industrial implementation. This review aims to highlight the importance of finding analytical techniques that can easily predict the quality of OO and, consequently, make more immediate assessments of its SL. In addition, the importance of SSL is intended to monitor post opening quality and thus avoid waste. This systematic review aims to critically analyze the available literature on sustainable analytical methods for assessing EVOO quality, paying attention to both technological and environmental aspects and economic implications, with a particular focus on reducing food waste. Non-destructive, rapid and sustainable techniques [such as Fluorescence Spectroscopy (FS), Nuclear Magnetic Resonance (NMR)] have the potential provide rapid information on quality and, consequently, on the SL of OO, responding to the marked need by the olive oil industries to develop efficient predictive models for determining SL (both PSL and SSL) and polyphenol claim validity.

## 2 Methods

**Figure 2:**
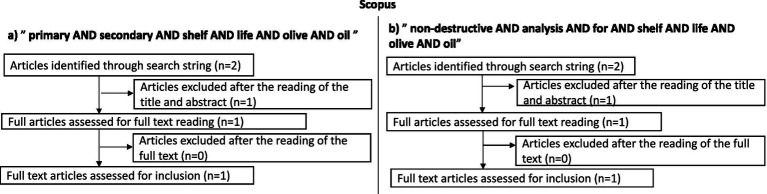
Flow charts illustrating the literature search and selection process conducted on the Scopus database. **(a)** Article selection using the search string “primary AND secondary AND shelf AND life AND olive AND oil” **(b)** Article selection using the search string “non-destructive AND analysis AND shelf AND life AND olive AND oil.” The diagrams show the number of records identified, screened, excluded, and finally included for the review.

**Figure 3:**
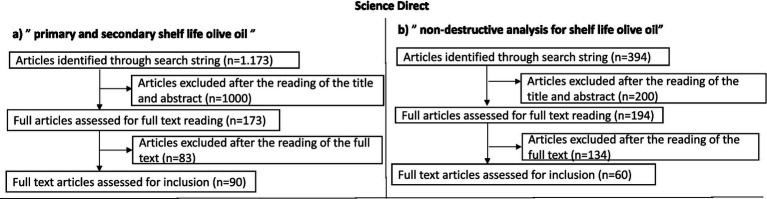
Flow charts illustrating the literature search and selection process conducted on the ScienceDirect database. **(a)** Article selection using the search string “primary and secondary shelf life olive oil”; **(b)** article selection using the search string “non-destructive analysis for shelf life olive oil.” The diagrams show the number of records identified, screened, excluded, and finally included for the review.

**Figure 4:**
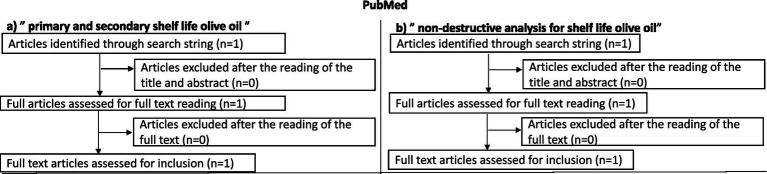
Flow charts illustrating the literature search and selection process conducted on the PubMed database. **(a)** Article selection using the search string “primary and secondary shelf life olive oil”; **(b)** Article selection using the search string “non-destructive analysis for shelf life olive oil.” The diagrams show the number of records identified, screened, excluded, and finally included for the review.

For the search strategy, information on olive oil production was retrieved from FAOSTAT, IOC, European Commission, different articles, and reviews. The research was focused mainly on scientific studies published since 2021, using the Scopus, PubMed, and Science Direct databases. The keywords were: “Virgin olive oil,” “Quality,” “Primary shelf life,” “Secondary shelf-life,” “Food waste reduction,” “Non-invasive methods,” and “Sustainable analysis.” When necessary, specialized websites, reviews, and book chapters were also consulted. The literature review explored the three databases using the following search string: “primary AND secondary AND shelf AND life AND olive AND oil” OR “non-destructive AND analysis AND for AND shelf AND life AND olive AND oil.” No restrictions were imposed regarding language or country of publication. Only a time restriction was applied, limiting the search to the last 5 years. The initial dataset was selected by reading the title and abstract (articles consisting only of an abstract and/or index were excluded at this stage) and then reading the full text. At the end of the selection process, all duplicates were excluded. All articles concerning any application of spectroscopy, NMR, and other non-destructive techniques to EVOO were included, as well as articles concerning both primary and secondary shelf life; those that did not deal with these topics were discarded. A flow chart was produced for each database to summarize the results obtained (Figures 2–4).

## 3 Results and discussion

The selected studies were 154 in total, involving articles, reviews, and book chapters. Ninety-two of them were relevant for discussing the quality parameters of EVOO that affect its shelf life, mainly primary and secondary oxidative phenomena that occur during storage time and that can be influenced by several intrinsic and extrinsic factors. Sixty-two were considered because they explored in depth the different analytical procedures, including non-destructive methods, which can be applied to the assessment of PSL and SSL. In this section, the main factors influencing SL will be described in order to characterize the mechanisms related to the loss of quality of OO.

### 3.1 Impact of environmental and intrinsic factors on shelf life of EVOO

SL of EVOO is mainly related to oxidative processes, which can be influenced by multiple environmental factors such as light, oxygen, and temperature; they are able to accelerate the formation of undesirable compounds in both the primary oxidation phase (e.g., hydroperoxides) and the secondary oxidation phase (aldehydes, ketones, volatile compounds) (18). These phenomena cause sensory and nutritional degradation, making the product no longer compliant with the required quality standards and leading to the product being downgraded by companies and premature disposal by consumers (19) contributing considerably to food waste (153). To address this issue, several studies have attempted to develop predictive models of EVOO SL based on chemical parameters such as K₂₃₂, K_270_ and pyropheophytin content, in order to estimate the actual SL of the product under real storage conditions. However, as highlighted by Ferreiro et al. (20) these models show considerable variability, depending on environmental conditions and the initial chemical profile. Added to this is the difficulty of ensuring the validity of the health claims reported on the label (e.g., polyphenol or vitamin E content), which often expire well before the stated expiration date (20, 21). The resistance of an EVOO to oxidation depends on its chemical composition and its exposure to pro-oxidant factors such as oxygen, light, temperature, and activators (chlorophylls and transition metals) during storage (22). Different studies have demonstrated that oxygen, light, and temperature are responsible for increasing deteriorative processes in EVOO as a consequence of oxidative and hydrolytic reactions (19, 23, 24). Oxidation can be counteracted by the antioxidant activity of polyphenolic compounds and tocopherols (25–30). The assessment of the SL of EVOO cannot ignore the role of packaging, which is a crucial element in preserving the chemical, sensory, and nutritional characteristics of the product. In particular, the PSL (still sealed package) and SSL (after opening the package) are strongly influenced by factors such as material permeability, exposure to light, and protection from oxygen (19).

#### 3.1.1 Effect of variety

**Figure 5:**
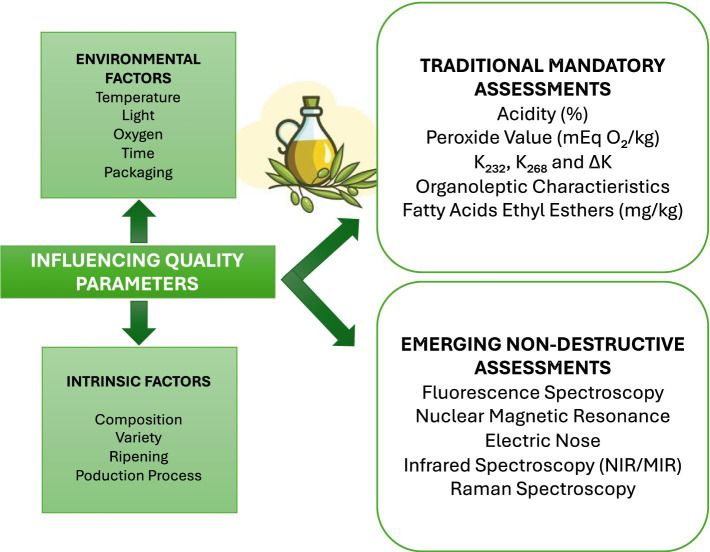
Factors regarding the quality influence of EVOO and overview of traditional and emerging analysis.

The final composition of EVOO is determined by the olive variety used, which constitutes an intrinsic factor influencing its stability and quality how showed in Figure 5. By accounting for variables such as olive cultivar, light exposure, and other conditions, producers can obtain EVOOs with varying concentrations of compounds like fatty acids, tocopherols, polyphenols, and other bioactive components. For example, Jimenez-Lopez et al. (31) showed an example of two varieties (Manzanilla Cacereña and Empeltre) in which the analyzes showed Manzanilla Cacereña OO contains a higher amount of pigments (chlorophylls and their derivatives such as pheophytins, as well as carotenoids), thus increasing the absorbance compared to the other variety (Empeltre). This affects EVOO coloration, since the greater the number of pigments results in a more yellowish greenish tonality. In addition, a higher density is also observed in the EVOO produced with the olives having the highest concentration of pigments (31). However, chlorophylls influence not only the characteristic color of EVOO but also its oxidative stability: in the absence of light, they can exert antioxidant activity, while when exposed to light, they can behave as pro-oxidants, thus accelerating photo-oxidation processes. This dual role highlights the importance of pigments concentration and storage conditions in assessing the overall quality of the oil (32).

#### 3.1.2 Effect of light, temperature, time, and oxygen content on EVOO storage

Light is one of the main factors that accelerate and promote the degradation of chemical compounds, such as phenolic compounds or pigments, responsible for OO quality. Esposto et al. (33) analyzed the effect of light in commercial varieties of EVOOs and showed that light has a greater degrading effect on those compounds that contribute to the health-promoting properties. The quantification of the different compounds (oleic acid, total phenolic compounds, fatty acids, tocopherols, and lignans) was carried out using HPLC, and showed that the loss of the “extra” category with time can be predicted depending on the initial concentration of oleuropein present in a certain EVOO (33). Another fast and efficient way to carry out light degradation analysis is by measuring pigments, such as chlorophyll (34). Therefore, light exposure should be avoided during the storage period to extend the SL of EVOO and preserve its properties (34). Oxidative processes and hydrolysis of the lipid substrate are more intense when EVOO is stored in the presence of light than in darkness or in presence of ions like Fe^2+^/Fe^3+^ (35, 36). Currently, fast and non-destructive analytical tools exist to check whether an EVOO has been correctly stored (22).

SL of OO is strongly influenced by storage temperature, a relationship confirmed by studies on diverse EVOOs (37). The ideal storage temperature is around 23 °C; while, higher temperatures (near 40 °C) can cause serious alterations in the saponified and unsaponified fractions. Alternatively, when EVOO is stored at low temperatures, a crystallization process caused by freezing can be observed. In addition, at low or moderate temperatures minor volatile compounds, in particular carbonyl compounds, are of enormous sensory significance and may contribute to modify the oil flavor and in consequence the final quality (31, 38, 39). Although temperature is the most common accelerating factor in shelf-life tests (ASLT), excessive heat (above 60 °C) is problematic. It can trigger oxidative processes and rancid off-flavors that do not reflect normal aging, leading to inaccurate SL predictions (40).

Another aspect that plays a key role in the quality and SL of the EVOO is the time. Generally, the SL of EVOOs is between 9 and 18 months, depending on other concomitant factors such as temperature or chemical composition. This finding supports the recommendation to consume EVOO as close as possible to its date of production (31, 41).

Key factors influencing lipid deterioration and, consequently, the overall quality and SL of EVOO during storage include its initial oxygen content after production, the oxygen permeability of the packaging, and the amount of oxygen in the headspace. A study conducted on the simultaneous effect of headspace oxygen concentration and presence of light on the quality of EVOO during storage time determined that the SL of EVOO could be maximized, exceeding 12 months of stability, by applying specific headspace oxygen levels between 2 and 5%. However, optimal preservation of beneficial compounds like pigments and polyphenols required storage in dark conditions, at low temperatures (around 10 °C), and with a headspace oxygen concentration limited to 2% (31). For this purpose, modified atmosphere packaging can be used, in which oxygen is replaced by other inert gasses such as argon o nitrogen (42).

#### 3.1.3 Effect of packaging material

Packaging selection is a critical factor influencing EVOO quality during storage. Common materials include tinted glass, polyethylene terephthalate (PET), tinplate, aluminum, Tetra Brik, and bag-in-box containers are summarized in Table 1. Packaging serves a functional role beyond containment, directly influencing the oil’s commercial and health value. The use of appropriate materials ensures an extended shelf life and safeguards the integrity of bioactive compounds, thereby enabling effective communication of their associated health benefits to the consumer. Many studies indicate that tinted glass is particularly effective for preservation; its inert nature prevents chemical interaction, while the tint protects the oil from light degradation (43). Polypropylene and polyethylene, on the other hand, due to their high oxygen permeability, are not recommended for long-term EVOO storage, as they fail to adequately protect the OO properties. Furthermore, a recent research indicated that bag-in-box packaging was more effective than tinplate containers at preserving EVOO and extending its shelf life under typical household storage conditions (22–37 °C) (31). Savarese et al. (44) investigated the changes occurring in bottled EVOO in different PET containers. The changes in quality indices, sensory parameters, phenolic antioxidants, and pigments of EVOO were evaluated in relation to prolonged storage (12 months). Another study evaluated two EVOO samples selected on the basis of their different acidity levels and phenolic contents and were bottled in transparent or red PET bottles. Bottles were placed in dark or light conditions at 25 °C, the latter simulating the normal conditions found in the supermarket. Lighting conditions were monitored using a digital chromatometer. During the night, bottles of EVOOs were exposed to a constant light intensity of 300 LUX provided by eight artificial neon lights, while daytime light intensity reached 500 LUX due to the effect of daylight. Results showed a significant decrease in the contents of *α*-tocopherol and chlorophylls during the first month of storage. Tocopherols seem to have a fundamental role in controlling and slowing down oil oxidation; in particular, α-Tocopherol showed a greater effect than phenolics in reducing photooxidation processes. The study of container characteristics revealed that red-tinted bottles offered inadequate light protection compared to transparent PET. Ultimately, effective preservation of the oil’s overall quality was achieved solely through storage in dark conditions. Results suggest the importance of finding containers with adequate barrier properties against light and oxygen to extend the SL of EVOO packaged in PET containers.

**Table 1:** Advantages and disadvantages of main packaging nowadays used.

Material	Advantages	Disadvantages	References
Glass (amber/dark green)	Barrier to oxygen and light Inert Easy to re-clean and re-use in order to go toward life cycle assessment (LCA) → eco-friendly system Gradual loss of quality during storage	Highly expensive to produce (heavy and breakable) Low resistivity to mechanical damage especially during transport and storage	(151)
Polyethylene terephtalate	Resistance to mechanical damage Economical, recyclable and lightweight Performable (adding nanoparticles like oxygen scaveners to prolong shelf life → Improve shelf life and quality during storage and reduce loss and waste on food) Better than polyvinyl chloride (PVC) and polyethylene (PE) for olive oil (OO)	No reusable → toxic component like bisphenol A (BPA) cuold be relased during reuse process Permeable material that allows the infliltration of gasses such as oxygen (O_2_)	(31, 43, 45, 151)
Tetra-brik	More efficient in maintaining the antioxidant compounds Better protection from light and oxygen compare to plastic containers, conserves the initial characteristics of the product much better Prolongs shelf life still to 2 years More efficient logistic, minor possibility of disruption during trasport	Not hugely used like container recognized by consumer for olive oil Very low acceptability by customers No reusable	(31)
Bag in box	Economical, lightweight Prolongs shelf life maintaining the quality for more time in terms of antioxidant compounds	No common container for package olive oil No reusable	(31, 151)
Smart, active and intelligent (nanosensors, tracers, indicators, etc.)	Improve barrier properties, shelf life and quality Allow to reduce loss and waste on food Has provided innovative approaches to improve food preservation	No actually degradation protocols to ensure the responsible use of nanotechnology in the food industry	(31, 45, 47, 152)

##### 3.1.3.1 Innovative packaging

Nowadays, nanotechnology is increasingly viewed as a key innovation for enhancing food quality and prolonging SL, positioning itself as a central element in the transition toward more sustainable food systems (45, 46). Chang et al. (45) examined the application of nanotechology in post-harvest practices and food packaging solutions with particular attention to nanocoatings, which contribute to maintaining the quality, and to nanoencapsulation techniques, designed for the controlled release of bioactive compounds. Both approaches play a crucial role in preserving the freshness of packaged foods. The same authors also explored active packaging technologies, particularly those aimed at oxygen scavenging, carbon dioxide regulation, moisture control, and antioxidant release, along with advancements in smart packaging, focusing on nanosensors, tracers, indicators, and monitoring devices. Finally, the discussion addressed safety and regulatory considerations, which are essential to ensure that these technologies meet consumer health requirements and environmental standards (45). Unlike conventional packaging, which serves as a passive barrier, smart packaging offers interactive functions like product tracking and real-time communication. While active packaging focuses on improving food safety and SL, smart packaging incorporates monitoring capabilities that provide timely alerts about product quality. A key category within this field is “intelligent” packaging, which uses elements like colorimetric indicators to give consumers direct visual signals about improper storage conditions (47). The defining characteristic of smart packaging lies in its ability to provide real-time data on food quality, including freshness, ripening, safety, and nutritional content. This innovation represents a shift from the traditional passive role of packaging toward systems that actively sense, interpret, and communicate changes in the surrounding environment (48). Nanostructured sensors are central to this transformation, as they enable monitoring of storage and transport conditions. These devices can detect variations in temperature and humidity as well as chemical compounds associated with spoilage, allergens, or toxins, thereby generating immediate information for corrective actions. In products such as EVOO, these advanced solutions can provide immediate feedback to producers and consumers on the internal conditions of the package during storage, thereby helping to reduce waste. Active packaging differs fundamentally from conventional systems in that it interacts directly with the food or the internal atmosphere of the package. Its purpose is not only to act as a physical barrier but also to modify storage conditions to enhance safety, extend SL, and improve sensory characteristics. Strategies include oxygen removal, moisture regulation, incorporation of antioxidants, antimicrobial action, and the integration of nanomaterials. Among these, oxygen control is particularly critical, as oxygen drives many deterioration processes such as lipid oxidation (49). Reducing oxygen inside the headspace of packaging should be an innovative initiative to extend the SL (45).

As an example, an application if innovative packaging of El Hamid and El-mahrouky (50) show how extra virgin olive oil (Coratina cultivar) packaged in Tetra Pak containers coated with edible films made from gelatin, gum arabic, and a gelatin-gum arabic compound affects oil preservation and therefore quality. The oxidative stability and quality of the olive oil were assessed by monitoring the number of peroxides, the free fatty acid content, and the concentration of bioactive compounds (in particular carotenoids and total polyphenols). In addition, the physical and mechanical properties of the edible films tested were characterized. The results showed that the application of edible films significantly prolonged the expected shelf life of EVOO compared to uncoated packaging (control). The gum arabic-coated packaging was found to be the most effective, providing a shelf life of 920 days, attributable to its intrinsic antioxidant activity and barrier properties against oxygen permeation. The composite film and gelatin film had shelf lives of 870 and 744 days, respectively, which were longer than the control (569 days). This demonstrates how edible films, particularly those based on gum arabic, can significantly preserve oxidative stability and extend the shelf life of EVOO. Furthermore, this experimental study highlights the potential of biodegradable edible coatings as a sustainable and effective alternative to conventional packaging, with significant implications for the development of environmentally friendly food packaging solutions for high-value lipid products.

Another study (51) evaluated the effectiveness of an active biodegradable film based on cassava starch and anthocyanins encapsulated with maltodextrin in maintaining the quality of EVOO. The oil was packaged in pouches made from the active film and compared with a commercial polypropylene film under accelerated degradation conditions (40 °C and exposure to fluorescent light). The main results showed that the active film effectively protected the EVOO, maintaining the peroxide value within the limits set by the Codex Alimentarius (≤ 20 mEq O_2_/kg) for over 8 days (reaching 13.58 meq O_2_/kg on the eighth day). In stark contrast, the oil packaged in polypropylene was highly degraded before the fourth day of storage (PV of 326.47 meq O_2_/kg on the eighth day).

Sadeghi et al. (52) developed and evaluated an active and intelligent biodegradable film based on cellulose nanofibers (Cel) modified with methylene blue (MB) and vitamin C (VC) for the packaging of virgin olive oil, with the dual purpose of acting as an antioxidant and a shelf life sensor. The oil was packaged in four variants, including the control and the Cel/MB/VC composite (B4), and stored for 30 days. The B4 packaging, in which MB was reduced by VC, initially appearing white, provided the best antioxidant performance, showing the lowest increase in acidity and peroxide value, and the greatest stability in total phenol content and sensory properties compared to the other samples. The smart function was confirmed by the Cel/MB/VC kit, which was able to estimate shelf life by changing color from white to blue depending on oil oxidation and storage time. A quantifiable linear mathematical relationship was obtained, allowing shelf life to be calculated accurately, which can also be detected using smartphone software.

Mignani et al. (53) present the design and validation of a micro-optic smart cap capable of non-destructively detecting the onset of rancidity in extra virgin olive oil by analyzing volatile compounds accumulated in the bottle headspace. The device employs an array of metalloporphyrin-based optochemical sensors, whose visible-range absorption spectra change in response to aldehydes (the key volatile markers of oxidative degradation). Spectral variations recorded during controlled artificial aging at 70 °C revealed progressive and reproducible changes in sensor transmittance, which correlated with increasing rancidity. Principal Component Analysis (PCA) of the 31 variable spectral dataset showed that a single principal component effectively tracked oxidation kinetics over time, enabling clear discrimination between fresh and oxidized states. While traditional assessment of oil degradation requires opening the bottle and performing destructive chemical or sensory analyses, the proposed UV–Vis-based smart cap allows continuous, real-time monitoring of oil quality without altering the product. The work demonstrates that optical interrogation of a sensorized cap can provide a reliable early warning of shelf-life deterioration, supporting its potential use in consumer packaging and quality control applications.

### 3.2 Relevance of accurate shelf-life assessment to reduce food waste

The SL of EVOO is defined as the period during which the product preserves its sensory characteristics (i.e., positive olfactory and gustatory attributes without sensory defects) and complies with regulatory quality parameters, including acidity, PV, specific extinction coefficients, fatty acid ethyl esters and wax content, under standard storage conditions (54, 55). Beyond these conventional quality indicators, the concept of SL may also encompass the persistence of specific nutritional or health-related claims (e.g., polyphenol concentration, vitamin E content, or the relative abundance of unsaturated fatty acids) when such information is displayed on the EVOO label. Determining the SL of EVOO is an important challenge for producers and is crucial for protecting consumers from food fraud. The provision of reliable SL information to consumers is essential; this depends critically on several factors, including packaging type, fluctuations in storage temperature, and exposure to light and oxygen at all stages of the supply chain. While recent studies show that SL varies widely (from 2 months to 2 years) based on factors like temperature, light, and oxygen (20). Other findings indicate that adequate protection from light and a reduced oxygen atmosphere (≤5%) can extend SL beyond 12 months (31). Furthermore, ideal storage conditions (filtration + refrigeration at 4 °C) can preserve the content of various organoleptic and chemical parameters for over 18–24 months (56). To regulate product classification, authorities have established individual analytical parameters to evaluate the quality and oxidative status of EVOO (57). However, due to the complexity of degradation, where multiple parameters are simultaneously involved and interact, there is a growing need for advanced multiparametric approaches to assess EVOO quality more accurately (58, 59). During distribution and retail, such variables can markedly alter EVOO stability, sometimes leading to discrepancies between the results of official control tests and the expiration date indicated on the label. This has raised concerns among regulatory agencies, which have issued guidelines for optimal storage practices (69) to mitigate rapid degradation. Nonetheless, these guidelines do not establish a maximum storage period, nor do they clearly define how to monitor oil deterioration over time. As a result, the analytical tools currently applied to ensure EVOO freshness and quality during storage remain limited and often ambiguous in interpretation. In practice, this lack of harmonized methodologies leads producers to define the SL of each batch according to their own internal criteria. Consequently, inconsistencies may arise between the actual quality of the product available to consumers and the quality stated on the label. The adoption of rapid, reliable, and innovative techniques could provide valuable solutions for more accurate quality control (60). SL evaluation may be performed either through real-time testing or via ASLT. ASLT often carries inherent limitations, as the extrapolated values may overestimate the actual stability of EVOO. These methodological constraints in ASLT have been well recognized for decades, underscoring the importance of real-time studies for obtaining more accurate and reliable predictions. Ultimately, analytical results from both real-time and ASLT approaches can be applied independently, or in combination, to construct predictive models for EVOO SL (20). Determining the SL of EVOO requires a structured, multi-step approach. The first step is the selection of a chemical, physical or biological parameter that reliably reflects the oil quality degradation or potential downgrading of its commercial category. Once the marker is identified, acceptable thresholds must be established, usually in accordance with regulatory limits for specific quality indicators (e.g., free acidity, PV, K_232_, K_268_) or for compounds linked to nutritional (e.g., minimum levels of unsaturated fatty acids or vitamin E per specified oil amount) or health claims (e.g., minimum polyphenol concentration) (20). The next step involves defining the storage conditions under which the analysis will be performed, either real-time storage, replicating conditions encountered along the commercial supply chain, or ASLT, which shortens experimental time by promoting degradation processes under stressed conditions. In real-time studies, oil quality is tracked under typical storage conditions. In contrast, ASLT uses a model of accelerated stability, which is generally conducted in three phases. The first phase involves measuring the degradation rates at elevated temperatures. Secondly, the relationship between temperature and degradation rate is established, usually following a logarithmic trend. Finally, this relationship is extrapolated to predict the SL of EVOO at desired storage conditions, typically at ambient temperatures (<30 °C). Predictive models are used to estimate SL by extrapolating experimental data. While effective, this method is inherently subject to uncertainty (20). Even though several methods are currently available to evaluate EVOO stability, these approaches rely on experimental conditions that differ substantially from those encountered during real storage. Such conditions alter the kinetics of oxidation and do not accurately reflect the degradation behavior of EVOO under moderate storage environments (39). Several modeling strategies have been described in the literature to estimate and predict the SL of EVOO. Most of these models are based on conventional approaches, namely the empirical-based model or the kinetic-based model (20); the latter is more widely applied (61). While empirical models often achieve high predictive accuracy, they typically rely on extensive chemical datasets, including markers without established regulatory thresholds and require advanced statistical analyses, which limit their practical use by non-specialists. By contrast, kinetic models provide simplified and more user-friendly mathematical equations, although their predictive reliability can still be influenced by variables such as initial oil composition, packaging material, and storage conditions. However, some literature reports propose a merged kinetic-empirical model (62) proposing. This highlights the need for continued refinement of modeling strategies to improve the robustness and applicability of SL prediction for OO (20, 63).

Despite considerable experimental and modeling efforts, the mathematical models are limited to kinetic and empirical approaches (64, 65). The empirical approach, in principle, can offer more comprehensive and realistic SL predictions, as it integrates a broad range of physicochemical and sensory parameters. Nonetheless, several critical limitations have been highlighted:

- *Extensive data requirements*: empirical models depend on large experimental datasets, often including detailed information on phenolic compounds, tocopherols, volatile compounds, and sensory attributes. Although highly informative, these data are not legally required for EVOO classification, making the approach both costly and time intensive. This complexity generally places it beyond the technical and financial reach of small and medium-sized producers.
- *Complexity of implementation*: empirical models are typically built using multivariate statistical techniques, which yield complex equations. These are difficult to apply in practice as routine tools for SL prediction, particularly for producers with limited resources.
- *Lack of regulatory support:* since empirical models often extend beyond legally defined quality parameters (such as those used for classifying EVOO, VOO or LOO, or for substantiating nutritional and health claims), they require the establishment of acceptability limits. While these thresholds are generally proposed on the basis of experimental data, they are not legally recognized, limiting the direct applicability of empirical approaches for commercial purposes.

In contrast, kinetic-based models are usually built on parameters with established legal thresholds, making them more straightforward to use in a regulatory context. However, they often rely on a single parameter, which can make them sensitive to the oil’s initial composition at bottling. Despite these limitations, the relative mathematical simplicity, reduced experimental requirements and the alignment with legal thresholds make kinetic-based approaches more practical and attractive for SL estimation (20).

#### 3.2.1 Kinetic approach

Conte et al. (40), investigated the stability of Italian monovarietal EVOOs (Coratina) stored in 250 mL transparent glass bottles, protected from light, and maintained at four different temperatures (25, 40, 50, and 60 °C) for a period of 300 days. The study monitored a wide range of quality parameters, including free acidity (FA, % oleic acid), PV (mEqO₂/kg oil), specific extinction coefficients (K232 and K270), total phenolic content, tocopherols, pyropheophytin-a, conjugated trienes and volatile compounds. To describe temporal changes, pseudo zero-order kinetic models were fitted to experimental data for K_232_, K_270_, and pyropheophytin-a, while the temperature dependence of lipid oxidation was successfully modeled using the Arrhenius equation, enabling the calculation of activation energies and frequency factors. The results showed that the estimated SL varied considerably depending on the chosen parameter, ranging from 32 to 377 days, with the shortest durations observed for K270 at 60 °C and the longest for K270 at 25 °C. Importantly, the study highlighted that PV and antioxidant levels were not suitable predictors of SL, whereas K270 proved to be the most reliable indicator. In addition, pyropheophytin-a was identified as a promising freshness index, showing greater sensitivity to temperature variations than K270, the conventional secondary oxidation marker (40). Calligaris et al. (66) examined the SL of EVOO produced in Italy in 2019, with oils classified into three groups according to their initial total polyphenol content (approximately 156, 273, and 507 mg/kg). The oils were obtained immediately after harvest and bottled within 1 month in 250 mL glass containers sealed with metal caps lined with polytetrafluoroethylene (PTFE) and leaving 2 cm of headspace. Samples were stored under conditions designed to replicate commercial practices, using incubators set at controlled temperatures (25, 40, 50, and 60 °C) in the dark for up to 300 days, with periodic sampling. SL estimation was performed using ASLT, with evaluation of common quality markers, including PV, specific extinction coefficients, total polyphenols, tocopherols and pyropheophytins (%PPP). The results showed that PV, K232, polyphenols and tocopherols remained largely stable under the tested storage conditions, never approaching their regulatory limits. This outcome aligned with the findings of Conte et al. (40). In sealed bottles, the reduced oxygen availability in the headspace limited the accumulation of primary oxidation products, thereby explaining the observed stability of PV and K232. In contrast, K270 and %PPP underwent notable changes, establishing them as the most reliable parameters for monitoring EVOO degradation during storage. SL prediction was conducted assuming pseudo zero-order kinetics for both indicators. Within this framework, the pyropheophytin index emerged as an early-warning marker for assessing market performance, while K270 was validated as a robust predictive indicator of SL (66). Li et al., (67) developed a kinetic model that considers both the formation and decomposition of lipid hydroperoxides during olive oil oxidation. The model tested by these authors was calibrated on experimental data obtained from olive oils subjected to accelerated oxidation conditions, in order to capture the dynamic behavior of hydroperoxide content over time. The proposed model showed R^2^ determination coefficients greater than 0.95. The results therefore support the suitability of the model for describing the dynamics of hydroperoxides in the context of olive oil.

#### 3.2.2 Empirical approach

Coutelieris and Kanavouras (64) highlighted some key aspects in predicting the shelf life of packaged olive oil using an empirical approach. The results show, first of all, that oxygen transport through packaging materials plays a negligible role compared to temperature and light. The model proved to be consistent and predictively reliable for samples stored in the dark, accurately describing the formation of oxidative compounds such as hexanal and allowing a comparative evaluation between materials with different barrier properties (e.g., PVC vs. PET). However, in the presence of light, the dominance of photo-oxidation significantly reduced the model’s ability to reproduce the qualitative evolution of the oil, highlighting a structural limitation of the approach. The analysis of this work confirmed the Arrhenius-type dependence of diffusion and highlighted a critical threshold around 15 °C, below which small increases in temperature cause a marked increase in the rate of oxidation. Overall, the approach offers a useful tool to support the selection of packaging materials and the definition of optimal storage conditions, despite the limitation of not being able to adequately describe the photo-oxidative mechanisms.

Di Serio et al. (65) investigated EVOOs obtained through a three-phase continuous centrifugation system from handpicked olives of seven cultivars (Biancolilla, Carolea, Coratina, Dolce di Rossano, Frantoio, Nocellara del Belice and Nocellara Etnea). The oils were packaged in 1 L dark green bottles, stored sealed at room temperature, and exposed to diffuse light, with average seasonal temperatures of approximately 15 °C in winter and 18 °C in summer. Samples were analyzed at bottling (0 months) and subsequently at two-month intervals up to 12 months. A wide set of parameters was monitored, including FA, PV, UV absorption indices, ethyl esters, 1,2-diglycerides, tocopherols, phenolic compounds, volatile compounds, fatty acid composition, sterol profile, and sensory attributes. The dataset obtained was then used to construct a mathematical model based on an empirical approach. Specifically, the relationship between oil age (Y_age_) and discriminant functions (Y_d_) was expressed through a single linear equation:


$$Y_{\mathrm{age}}=(a\times Y_{d})+b$$

The discriminant function Y_d_ was calculated as a weighted linear combination of independent variables selected by the algorithm. EVOO samples were grouped by storage time (0, 2, 4, 6, 8, 10, and 12 months), with each group containing one independent oil from each cultivar. Linear discriminant analysis (LDA) revealed that the first discriminant function explained the majority of variability in the dataset and was therefore proposed as the Y_d_ function. Using this model, they demonstrated that the age of unknown EVOOs could be predicted with an accuracy of ±1 month. This approach also allowed estimation of the remaining storage time, assuming a typical SL of approximately 11 months for EVOO. Nevertheless, the authors emphasized that the reliability of this model depends on a comprehensive set of physicochemical data, making the procedure labor-intensive and costly. As a result, its application may not be practical for small-scale olive oil producers (65).

### 3.3 Secondary shelf life

**Figure 6:**
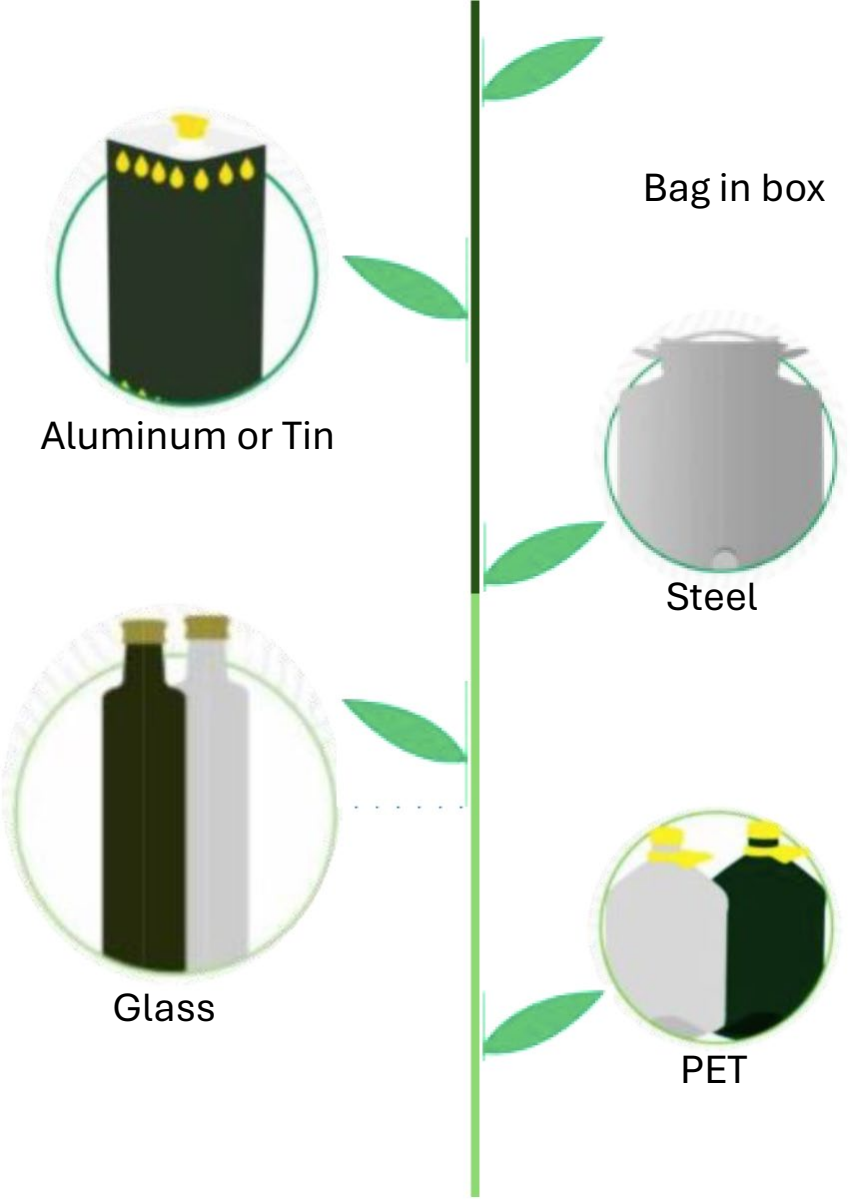
Different packaging that may influence the quality and SSL of EVOO.

The SSL of foods plays a crucial role in determining the actual quality perceived at the point of household consumption. Upon opening, food is subjected to significant environmental shifts, including temperature change, disruption of the modified atmosphere, increased oxygen availability, exposure to ambient humidity, and potential microbial contamination, that accelerate its degradation (68). Consequently, SSL is typically much shorter than PSL and its management largely depends on consumer practices. Inadequate handling of SSL can lead to premature quality deterioration and increased household food waste. Critical factors include the package opening time, storage temperature, frequency and manner of resealing and portion removal, all of which affect the quality delivered at the consumption stage. This variability complicates the establishment of SSL guidelines. Nevertheless, food industries are increasingly required to provide indications of SSL and storage recommendations on packaging labels, ensuring both safety and quality of consumption (68). Some packaging materials provide enhanced protection against external factors such as oxygen, moisture, or microbial contamination even after the seal is broken, thereby extending SSL. Developing reliable assessment methods is therefore essential, both to guide consumer behavior and to minimize waste and health risks. Despite its relevance, SSL remains a largely underexplored research area; there are no standardized methodologies or clear regulations for SSL determination. Expanding research in this field would provide valuable insights for consumers, while also helping the food industry refine storage strategies and comply with increasingly strict food safety and quality regulations. The IOC (International Olive Council) has also drawn up guidelines for consumers and in 2020 issued a document providing them with useful information on storing EVOO after opening the original container, again to limit oxidation as much as possible and reduce waste (69, 70). Therefore, monitoring the quality of VOO throughout its SL represents a major challenge for the OO sector, reinforcing the need for smart packaging systems capable of tracking quality in real time especially about SSL (71). Currently, there are several types of packaging that can affect the SSL of EVOO (Figure 6). SSL is influenced primarily by the properties of the packaging material and by the storage conditions applied post opening (72, 73). Further factors influencing post-opening stability are the frequency of use (which escalates exposure to oxygen, light, and thermal stress), the cumulative time the product remains exposed at ambient temperature, and its inherent quality at the time of opening (74–76). Among secondary storage conditions, temperature plays a particularly critical role. For example, Krichene et al. (77) evaluated the stability of four VOO cultivars during extended storage (up to 18 months after first opening) at different temperatures (5, 15, 25, and 50 °C). Their findings confirmed that storage at temperatures below the typical commercial range (20–25 °C) enhanced oil preservation. Beyond the established influence of secondary packaging and storage conditions, there is currently a lack of standardized protocols or systematic approaches to reliably predict SSL. In this regard, attention should also be directed toward the extent of deterioration reactions already occurring at the moment of package opening, as these may critically affect subsequent SSL. Only a limited number of studies have addressed this issue. Products consumed over extended periods after opening, such as roasted coffee, infant formula powders, wines, and sauces, have been the main focus of SSL investigations (72, 78–80). Most of these studies rely on experimental evaluations simulating consumer use, while a stronger theoretical basis for SSL management remains under development. Cesa et al. (81) investigated infant formula, characterized by a long PSL of about 2 years, under different storage conditions and varying opening times. Their results highlighted not only the significance of post opening storage, but also the impact of the degradation state of the product at the time of unsealing (81). Likewise, Nicosia et al. (75) demonstrated in UHT milk that SSL was longer when the package was opened shortly after commercialization, whereas products opened toward the end of their PSL exhibited shorter SSL (75). This review highlights the need for further research aimed at developing comprehensive mathematical models based on food deterioration kinetics, capable of describing the interplay between package opening time, quality degradation rates, and SSL. Such models could provide a theoretical framework to support improved SSL management, enhance food quality at the point of consumption, and contribute to the reduction of food waste across the supply chain. Understanding and exploiting SSL can offer several advantages. It would allow for a more accurate understanding of the product’s actual durability: many companies rely solely on a precautionary estimate of the minimum SL, but the oil could retain its properties for longer if stored correctly. Having data on post-opening SL would allow for better calibration of the product life cycle. In addition, companies would be able to choose more functional packaging materials (e.g., dark glass, measuring caps with oxygen limiters) and improve product transport and storage. Therefore, suitable tools for the SSL determination would present a dual-benefit framework, enhancing value for both consumers and producers within the olive oil supply chain. For consumers, SSL provides data-driven, reliable information to mitigate the premature disposal of edible oil, thereby directly addressing household food waste. For olive oil companies, the application of real post-opening stability data facilitates the optimization of production and logistical planning. Furthermore, it enables the implementation of smart labeling solutions (such as dynamic QR codes, indicating personalized best-before dates), which concurrently advance product traceability and market transparency. Ultimately, SSL transforms static shelf-life estimates into a dynamic tool for operational efficiency, waste reduction, and consumer empowerment (82).

### 3.4 Emerging non-destructive analytical techniques

EVOO quality is currently assessed using physicochemical parameters and sensory analysis following IOC and Codex Alimentarius standards. However, existing methods inadequately evaluate quality deterioration during storage and fail to capture complex alteration processes affecting beneficial and sensory properties (83, 84). Furthermore, the current analytical methods are destructive, time-consuming, require skilled operators, use toxic solvents, and generate conspicuous environmental costs. They are also incompatible with real-time industrial analysis. Therefore, rapid, non-destructive, and sustainable analytical strategies (i.e., electronic nose, spectroscopy, and NMR) are needed to ensure EVOO quality while reducing food waste and supporting environmental sustainability (85–89, 154). Non-destructive emerging techniques applied on food are summarized in Table 2. Through real-time analytical capability, these techniques permit the early identification of deviations from quality standards and supports prompt adjustments, ensuring that final products consistently meet both consumer expectations and regulatory requirements (85). Li and Wang (90) analyzed several predictive models to estimate the shelf life of EVOO, highlighting how certain non-destructive techniques (NIR, MIR, etc.) based on spectroscopy and electronic sensors can monitor oxidative changes without resorting to invasive procedures or those requiring hazardous solvents.

**Table 2:** Overview of non-destructive techniques applied on EVOO.

Analytical techniques	Aim of the study	References
Fluorescence spectroscopy	EVOO quality assessment during aging	(38)
	Determination of origin of different French virgin olive oils (traceability purposes)	(111)
	Characterization and authentication of extra virgin olive oils	(109)
	Control virgin olive oils quality	(91)
	Characterization and provide fingerprint of olive oil	(112)
	Monitoring oxidation stability and qualitative decay of antioxidants in EVOO	(27)
	Shelf-life assessment of virgin olive oil	(60)
	Discriminating two different EVOOs according to age, variety and irrigation conditions	(24)
	Detection of the presence of refined hazelnut oil in refined olive oil	(110)
	Determination of overall olive oil quality characteristics (tocopherols, phenolic compounds and chlorophylls)	(108)
	Monitoring of extra virgin olive oil during storage	(71)
	Chemical changes of thermoxidized virgin olive oil	(114)
	Extraction of physicochemical properties of olive oil for quality control	(115)
	Extraction of physicochemical properties of olive oil for quality control	(104)
NMR spectroscopy	EVOO Storage stability assessment	(22)
	Fatty acids characterization of sunflower, olive and linseed oil	(121)
	Quality assessment of olive oil and other vegetable oils	(86)
NIR spectroscopy	Estimating the α–tocopherol and total tocopherols contents on olive oil	(128)
	Assess quality and authenticity of olive oil	(101)
	Identification of geographical origin of olive oils	(127)
	Detection of olive oil adulteration	(130)
	Estimate polar phenolic profile, quality and commercial grade of VOO	(29)
	Classification of extra virgin olive oil on the basis of fruity attribute intensity	(124)
FTIR spectroscopy	Real time monitoring of the combined effect of chlorophyll content and packaging of virgin olive oil	(34)
UV–Vis spectroscopy	Detection and quantification of the addition of vegetable oils into olive oil	(132)
Innovative approaches	Olives and olive oil quality assessment	(105)
	Assess stability of Arbequina EVOO	(58)
	Merging information on characterization and assessment of olive oil	(59)
	Authentication and quantification of adulteration in EVOO	(139)
	Quality assessment of olive oil studying the effects of thermal degradation intended as accelerating aging condition	(103)
Raman spectroscopy	Monitoring carotenoids during thermal aging of olive oil	(144)
E-nose	EVOO quality assessment during storage	(147)

It is also worth noting that the combination of chemometric and spectroscopic techniques to develop methods in line with the concept of green chemistry has become an important challenge in recent decades (91). For example, given the complexity of lipid oxidation pathways in EVOO, it is crucial to establish correlations between representative oxidation products and appropriate analytical methods (92).

**Figure 7:**
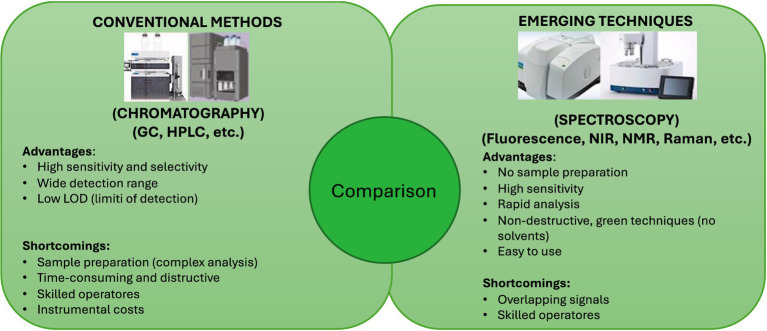
Difference between destructive chemical analysis and emerging analytical techniques to evaluate the quality of EVOO (advantages and disadvantages).

Recent literature has highlighted both advantages and limitations of classical and modern approaches. Zhang et al., (93) compared titration-based methods with instrumental techniques for determining PV, emphasizing their current challenges and perspectives. In parallel, they reviewed oxidation mechanisms and summarized the main indices, along with chromatographic and spectroscopic approaches for oil analysis. Conclusion of this study shows that the traditional destructive titration method for peroxide value determination remains accurate and officially accepted, yet it is limited by the use of hazardous solvents such as chloroform, along with its time-consuming and reagent-dependent nature. In contrast, non-destructive Fourier Transform Infrared (FT-IR) and Near-Infrared (NIR) techniques offer rapid, reagent-free analysis and preserve the sample, making them attractive for routine quality monitoring. However, their broader application is hindered by calibration challenges: NIR models require extensive, geographically representative datasets, while FT-IR measurements can be affected by oil viscosity and therefore rely on specific procedures or accessories to ensure reliability. Overcoming these limitations would significantly enhance the adoption of FT-IR and NIR as alternatives to titration for monitoring oxidation in edible oils. Despite the wide range of available techniques, no comprehensive overview has yet been provided on the applicability, accuracy, and feasibility of the different analytical strategies, particularly regarding rapid detection methods (94). Lu et al. (95) provide an updated synthesis of analytical methods for the determination of oil oxidation, discussing their detection principles, instrumentation, and practical feasibility. Recently, spectroscopic methods such as NIR and FT-IRhave gained prominence for edible oil evaluation due to their speed and environmental friendliness (96, 97). However, recent innovations in vibrational spectroscopy, particularly NIR and Fourier Transform Near-Infrared (FT-NIR), have enabled accurate PV assessment with minimal sample preparation (97). Similarly, Hafer et al. (98) introduced the ^1^H-(^31^P) decoupled NMR approach, which provides rapid and precise PV determination, offering manufacturers a valuable tool to safeguard SL and ensure product freshness. As an alternative example, Pulassery et al. (99) demonstrated that handheld Raman devices can accurately estimate values based on the ratio of the C=C stretching band (1,658 cm^−1^) to the CH₂ bending mode (1,442 cm^−1^), yielding results comparable to conventional assays. Similarly, FT-IR combined with chemometric modeling has proven effective in refining predictions, underscoring the potential of advanced spectroscopic tools to streamline quality control while reducing environmental and operational burdens. FT-IR spectroscopy has been shown to be particularly effective in tracking EVOO quality changes by monitoring the lipid oxidation under accelerated storage conditions, which is essential for predicting the longevity and stability of edible oils (100). Vibrational spectroscopy methods like NIR, IR and Raman Spectroscopy provide molecular ‘fingerprints’ that reveal critical information about oil samples in a matter of seconds without handling of the samples (85). These methods enable rapid quality and authenticity assessments by oil processors, while enabling regulatory agencies to verify labeling claims efficiently. Additionally, these techniques preserve sample integrity, making them well-suited for continuous, in-line quality monitoring during production processes. Casale and Simonetti (101), confirmed that NIR emerges as a rapid and versatile tool for quality control, process monitoring and estimating the SL of OO. Predictive models based on NIR show good performance: for example, for acidity and peroxide value (R^2^ ~ 0.88), with minimal exposure to destructive techniques (102). Recent studies show that FS, combined with machine learning (ML), allows for non-destructive monitoring of EVOO oxidation during storage, in a cost-effective manner and even in the field (103). An approach using portable fluorescence sensors and 1D-CNN algorithms was also developed to predict quality parameters (acids, peroxides, K₂₃₂, K₂₇₀, ethyl esters), demonstrating excellent accuracy (104). The literature widely supports the use of non-destructive techniques such as NIR, FS, and NMR to monitor and predict oil quality and SL (2, 91, 102, 104, 105). Although the direct link with packaging is still an emerging field, these techniques could also become excellent tools for evaluating how different kinds of packaging preserve the bioactive compounds, especially in the post-opening phase (SSL). Summing up, the advantages and disadvantages of conventional and emerging techniques for the shelf life analysis of EVFOO are compared in Figure 7.

#### 3.4.1 Fluorescence spectroscopy

The necessity of controlling a wide range of parameters throughout the food supply chain, particularly during storage, highlights the potential of spectroscopy as a suitable technique for providing comprehensive information on food quality. Spectroscopic methods have been widely adopted in food analysis because they allow rapid, efficient, and non-destructive measurement of numerous chemical constituents in complex food matrices (60, 106). Spectroscopy techniques, such as NIR and mid-infrared spectroscopy (MIR), FT-RS and FS, offer promising results with minimal samples, simultaneous analysis, and excellent repeatability, representing a valuable alternative to classical analytical methods, and their use in the analysis of edible vegetable oils is expanding rapidly (83, 107). FS technique provides highly sensitive detection of fluorescent compounds in vegetable oils, at lower concentrations than those detectable with absorption spectroscopy (108). The acquisition of an excitation–emission matrix (EEM), a three-dimensional spectrum or contour map containing signals from all fluorophores in the sample, enables comprehensive profiling of fluorescent compounds. Multivariate analysis of EEM data allows simultaneous extraction of information on the different fluorophores present in a food system. This makes FS particularly well suited for studying EVOO, a complex matrix that contains multiple fluorescent components such as phenols, tocopherols, aromatic compounds, and pheophytins.

Beyond quality and characterization assessment, it has also been applied to detect adulteration, such as blending EVOOs with other vegetable oils (109, 110), discriminate oils according to their geographical origin (111, 112), and rapidly assess the degradation of quality during the storage by using predictive models. Recent studies have proposed chemometric models based on fluorescence signals to estimate the oxidative state in oils stored under different conditions (113). In addition, applications have been developed that use total synchronous FS and excitation-emission fluorescence spectroscopy (EEFS) to monitor changes in EVOO during storage under different conditions and in the chemical composition of thermo-oxidized virgin olive oil (114). Venturini et al. (115) proposed a fast method based on one single fluorescence spectrum, obtained by a portable fluorescence sensor and coupled to one-dimensional convolutional neural networks (1D-CNN), to predict five chemical quality indicators of olive oil (acidity, peroxide value, UV spectroscopic parameters *K*_270_ and *K*_232_, and ethyl esters)for the determination of the SL of EVOO. Despite being trained on a relatively small dataset, the method returned reliable predictions with accuracy comparable to typical experimental uncertainties. It is worth pointing out that this new approach required low-cost instrumentation, no sample preparation, enabling on-site use by non-experts, and could be applied to other substances, underscoring the broader promise of 1D-CNNs for rapid spectral analysis.

The study by Lobo-Prieto et al. (60) investigated the application of FS, combined with total luminescence and PARAFAC chemometric analysis, to monitor shelf-life and quality degradation in virgin olive oil (VOO) under simulated market storage conditions. These conditions included light/dark cycles, moderate temperature, and humidity. Over 21 months, four monovarietal VOOs were stored in transparent PET bottles and analyzed monthly. The researchers compared EEMs against conventional quality markers, concentrations of key fluorescent compounds (e.g., phenols, tocopherols, chlorophyll derivatives), and sensory attributes (fruitiness, defects) assessed by a trained panel. The findings demonstrate that FS-PARAFAC constitutes a robust, multiparametric, and sensitive method for tracking VOO shelf-life under realistic storage scenarios. While conventional chemical and sensory evaluations often capture only isolated or late-stage degradation, FS offers an earlier and more integrated indicator of quality loss. This approach holds potential utility for producers, quality control agencies, and possibly regulatory bodies.

Guzmàn et al. (116) by applying multivariate techniques built predictive models linking fluorescence spectra to conventional quality parameters. This research showed that FS, especially when combined with chemometric analysis, offers a promising alternative or complement to classical analytical methods for evaluating olive oil quality. The best prediction performance was achieved for K₂₇₀ (which reflects secondary oxidation) with an external-validation correlation coefficient of ~ 0.924. Because FS is non-destructive, fast, and inexpensive (no reagents or extensive sample prep), it could support routine quality control, enabling producers, processors, or regulatory bodies to assess oil quality more frequently and cost-effectively.

#### 3.4.2 Nuclear magnetic resonance spectroscopy

Fingerprinting methods such as NMR are particularly attractive since they are non-selective, require little or no sample pre-treatment, use small amounts of organic solvents or reagents, and are typically less time-consuming, allowing high and fast throughput analysis (22). The method exploits the magnetic properties of atomic nuclei such as ^1^H, ^13^C, and ^31^P, which exhibit non-zero spin angular momentum and associated magnetic moments. When subjected to a strong magnetic field, these nuclei align either parallel or antiparallel to the field, generating a net magnetization (117). Radio-frequency pulses applied at the Larmor frequency cause transitions between spin states and the resulting relaxation processes produce RF signals that are transformed into NMR spectra. These spectra provide highly detailed information on molecular structures, chemical environments and dynamic processes within the sample (117). Proton nuclear magnetic resonance (^1^H NMR) spectroscopy has emerged as a powerful analytical tool, providing a unique “fingerprint” of each EVOO sample (22). Through multivariate analysis (MVA) of ^1^H NMR spectral data, the temporal evolution of EVOO was characterized, identifying the chemical compounds most responsible for compositional changes resulting from hydrolytic and oxidative degradation. Based on these data, classification models were developed to distinguish between fresh and non-fresh oils and to verify the extent of exposure to light during storage. Additionally, regression models were constructed to estimate EVOO age, providing insights into its effective storage duration and, preliminarily, the expiration date of fresh EVOO. These findings contribute to improving quality control and storage management strategies for the olive oil industry (22). In addition to the practical advantages, NMR is capable of simultaneously analyzing multiple target analytes (fatty acids, triglycerides, sterols, diglycerides, and oxidation products) through quantitative analysis in rapid time, without any internal standards (118, 119). ^1^H NMR can provide data closely related to the evolution of the classical parameters, such as PV, conjugated dienes, etc., used to evaluate the oxidation stability of EVOO. Therefore, synergisms among oil components in relation to oxidation have been observed. Despite challenges such as relatively low sensitivity, high instrument cost, the need for specialized expertise, and the complexity of data interpretation for large biomolecules, technological advances have alleviated many of these limitations. The introduction of benchtop low-field NMR systems (43–60 MHz) and high-resolution magic angle spinning (HR-MAS) has reduced operational costs, minimized sample preparation, and facilitated the study of heterogeneous or semi-solid matrices. Low-field benchtop instruments have been shown to detect oil adulteration and determine fatty acid composition with accuracy comparable to that of high-field systems, while HR-MAS extends applicability to more complex food systems (85). The integration of chemometrics and ML further enhances the potential of NMR, allowing robust classification, authentication and predictive modeling in food analysis (120). Importantly, these approaches have also enabled the use of NMR for predicting product SL, by monitoring degradation pathways and compositional changes that occur during storage. This makes NMR not only a tool for structural characterization and authenticity testing, but also a reliable method for forecasting the stability and longevity of food products under commercial conditions (22). Recent progress in NMR has further strengthened analytical capacity in EVOO studies, such as the automated method proposed by Castejón et al. (121), who have considerably reduced acquisition times to approximately 3 min, markedly improving throughput in routine assessments (121). Low-field NMR has demonstrated effective performance in fatty acid quantification, making the technique more accessible for widespread industrial use and compliant with EU labeling requirements. Hoppenreijs et al. (100) use ^1^H NMR to test accelerated shelf life of oil blends. In this study is reported that analytical procedures confirmed that the hydroperoxide and aldehyde concentrations quantified by ^1^H NMR were consistent with the hydroperoxide and para-anisidine values obtained through conventional spectrophotometric destructive methods, thereby demonstrating the reliability of the NMR-based approach. Alonso-Salces et al. (122) used ^1^H NMR to identify oxidative products from oxidative processes that occur on olive oil in order to evaluate oxidative stability of the product. This study provides proof-of-concept evidence that ^1^H NMR represents a robust alternative to destructive analytical methods for assessing the oxidative stability of edible oils. The technique enables reliable monitoring across a wide range of temperatures and offers the additional advantage of detecting and quantifying potentially toxic compounds formed during degradation, underscoring its value for comprehensive quality control. Furthermore, Merkx et al. (123), demonstrated that conventional methods such as the peroxide value, titration and headspace gas chromatography (HS-GC) provide only partial and indirect insights into lipid oxidation, as they quantify total hydroperoxides or single volatile aldehydes. By contrast, quantitative ^1^H NMR offers a comprehensive, simultaneous, and structurally detailed assessment of both primary and secondary oxidation products. The authors optimized a band-selective ^1^H NMR approach that enabled sensitive detection and precise quantification of multiple hydroperoxide and aldehyde species. Comparison with peroxide value showed no significant systematic error, demonstrating that ^1^H NMR provides equally accurate quantification of hydroperoxides, and its correlation with HS-GC hexanal measurements confirmed its robustness in tracking secondary oxidative products. Overall, the results show that ^1^H NMR is not only equivalent to traditional methods in quantifying key oxidation markers but is substantially more informative and efficient for evaluating oxidative stability during storage, providing a multidimensional and mechanistically sensitive tool for shelf-life assessment.

#### 3.4.3 Infrared spectroscopy—Fourier transform mid-near infrared spectroscopy

IR spectroscopy is a widely used analytical tool for the safety and quality evaluation of edible oils, providing detailed insights into their molecular structure and composition. The fundamental principle of IR spectroscopy involves the interaction of infrared light with the vibrational modes of chemical bonds within oil constituents. When molecules absorb IR radiation, their chemical bonds undergo stretching, bending, or twisting motions based on the specific vibrational mode and the energy of the absorbed light. Functional groups such as carbonyl (C=O), hydroxyl (O-H), and carbon-hydrogen (C-H) bonds exhibit unique absorption bands in the IR spectrum, facilitating their identification and quantification. IR spectroscopy is categorized into NIR and MIR regions, each capturing different vibrational aspects. NIR spectroscopy (750–2,500 nm) primarily measures overtones and combination bands of fundamental molecular vibrations, making it suitable for high-throughput screening and online monitoring due to its efficiency and cost-effectiveness. Thanks to its rapid, accurate, simple, and economical method for evaluating chemical constituents, IR spectroscopy in combination with chemometrics is one of the non-destructive tools widely used by the food and beverage industry for quality testing and analysis (124). These techniques enable efficient analysis and allow the simultaneous quantification of multiple analytes. Its versatility is evident in its wide range of applications in food quality and safety evaluation, including compositional analysis, adulteration detection and quality control (125). FT-IR spectrometers offer superior speed and sensitivity, having several important advantages over dispersive IR spectrometers, such as a better signal-to-noise (STN) ratio of the spectrum compared to previous generation infrared spectrometers. FT-IR spectrometers have higher wavenumber accuracy, a low error range (±0.01 cm^−1^), short scanning time (approximately 1 s), and high resolution (0.1–0.005 cm^−1^). In combination with chemometric tools, both FT-NIR and FT-MIR have several advantages and limitations. For example, FT-NIR has inexpensive components due to the use of low-cost materials such as glass and quartz compared to FT-MIR. FT-NIR also uses more robust components and is easier to manufacture into robust instruments with no moving parts. FT-MIR, on the other hand, contains more spectral information due to the higher resolution of fundamental vibrational absorption bands and can identify very complex or similar structures compared to the broad harmonic and combined absorption bands in the NIR region (126). Another advantage of FT-MIR is that it includes the fundamental vibrations of molecular bonds within a sample that occur in the “fingerprint” region, making spectral profiles very sensitive; even very similar molecules can produce quite distinct spectral bands. As a disadvantage, the absorption bands of the FT-MIR spectra are very broad and overlap due to the presence of numerous chemically different samples, resulting in almost indistinguishable spectral profiles.

FT-IR spectroscopy has been successfully used to classify geographical locations and sources of oils and detect adulteration (127). Some of the main attributes accessible via IR spectroscopy include phenolic content, carotenoid content, PV, yellowness index and fatty acid composition in different oil products, including EVOO (128). The development of methods combining FT-IR spectroscopy and chemometrics has the potential to provide new insights into the non-destructive prediction of oil quality for both authentication and adulteration. In fact, a study of Okere et al. (129) investigated the feasibility of using FT spectroscopy in the NIR and MIR regions for evaluating kernel oil quality. This was performed both qualitatively, using principal component analysis (PCA) and orthogonal partial least squares-discriminant analysis (OPLS-DA), and quantitatively, via partial least squares (PLS) regression. Advancements such as portable NIR devices have expanded their applicability from laboratory to field and industrial environments, enabling on-site quality control and adulteration detection (85, 130). Vibrational spectroscopy is an analytical technique that studies the interaction between electromagnetic radiation and molecular bond vibrations. Each chemical bond has a unique vibrational energy, resulting in distinctive spectral fingerprints for molecules. Recent advancements in FT-IR, including the integration of attenuated total reflectance (ATR) sampling accessories and novel sample preparation methods, have enhanced its sensitivity and versatility for analyzing liquid and semi-solid oils. The integration of IR spectroscopy with advanced data analysis has improved edible oil analysis by precisely interpreting complex spectra (85). Despite these advancements, IR spectroscopy faces challenges such as signal interference from overlapping bands, matrix effects, and the presence of minor components, which can impact measurement accuracy and reliability. Ongoing research is focused on developing more robust calibration models, optimizing sample preparation techniques and exploring novel data pre-processing methods to mitigate these limitations (85). As the demand for quick, reliable, and sustainable analytical methods grows, IR spectroscopy is set to play an increasingly vital role in ensuring the safety, quality, and authenticity of edible oils. Future trends include the integration of IR spectroscopy with artificial intelligence and deep learning (DL), which promise to further enhance the accuracy and efficiency of edible oil quality assessments (85).

Kharbach et al. (131) employed FT-IR spectroscopy coupled with chemometrics and conventional chemical assays to monitor the shelf-life of extra virgin Argan oil over 2 years under normal storage conditions. Using PCA and partial least squares-discriminant analysis (PLS-DA), they successfully distinguished fresh from oxidized oils based on both chemical parameters and FT-IR spectra. Furthermore, PLS regression models applied to the FT-IR data accurately predicted key physicochemical indices throughout storage. This confirms that mid-infrared spectral fingerprints contain sufficient information to approximate standard quality metrics. The ability of FT-IR-chemometrics not only to classify samples but also to predict the progression of degradation suggests this approach is transferable to other high-value oils, such as extra virgin olive oil, for industrial or regulatory shelf-life estimation. In summary, the study demonstrates that mid-infrared spectroscopy, when integrated with chemometric analysis, can serve as an efficient, non-destructive surrogate for traditional chemical testing in monitoring oil degradation, supporting its broader application for rapid and scalable quality control. Tena et al. (155) applied a mesh cell accessory to store monocultivar virgin olive oils under a range of moderate temperature (23, 35, 65 °C) and light conditions, simulating typical storage and transport environments. Using FT-IR spectroscopy, they monitored oxidation in real time and compared results with standard chemical assays. The FT-IR spectral changes revealed the sequential formation of primary oxidation products (hydroperoxides) and subsequent secondary products (alcohols, aldehydes), even under mild, non-accelerated conditions. This holistic spectral fingerprint provides a more comprehensive stability assessment than single-parameter chemical tests. FT-IR demonstrated sensitivity to early oxidative changes (e.g., detecting hydroperoxides before significant rises in conventional indices) while also tracking the progression to secondary products linked to sensory defects (off-flavors) and shelf-life reduction. Consequently, the authors propose that FT-IR enables the differentiation of oils based on oxidative resistance under realistic storage conditions, offering insights that traditional chemical parameters may fail to capture.

#### 3.4.4 Ultraviolet–visible (UV–vis) spectroscopy

UV–Vis is one of the most established analytical tools in the food sector, particularly for the characterization of edible oils. This technique measures light absorption in the 200–800 nm range, capturing electronic transitions in unsaturated compounds (132). As such, it is particularly effective in detecting unsaturated fatty acids, pigments and antioxidants by identifying functional groups such as phenolics, conjugated double bonds and aromatic rings, thereby providing valuable information on the chemical composition and concentration of compounds within food matrices (133). Its main advantages include simplicity, rapid analysis, low cost, and fast data acquisition. UV–Vis spectroscopy could be insufficient in terms of sensitivity for detecting trace compounds, although this drawback can be mitigated by appropriate sample preparation techniques (134). Recent technological improvements have sensibly expanded the application of UV–Vis spectroscopy in edible oil analysis. Dual-beam spectrometers, for instance, enhance stability and accuracy by simultaneously referencing sample and baseline measurements, while high-intensity deuterium and tungsten-halogen lamps ensure consistent illumination across both UV and visible regions (85). These advances enable precise analysis of complex samples, even at low concentrations. High-resolution and rapid-scanning monochromators also facilitate more accurate wavelength selection and real-time data acquisition. Complementary advances in Vis–NIR spectroscopy also allow accurate detection of vibrational modes associated with fatty acids, as demonstrated by Su et al. (135) in the quantification of palmitic, stearic and arachidic acids, thereby reinforcing its utility in comprehensive quality control. Moreover, the development of portable and miniaturized UV–Vis instruments has opened new opportunities for on-site applications in environmental monitoring and quality control across the edible oil supply chain. Fiber-optic probes now allow remote sampling, while photodiode arrays (PDAs) improve sensitivity and reduce noise for the detection of trace compounds. In parallel, advancements in software, particularly chemometrics and machine learning, have enhanced spectral interpretation and predictive modeling (133). Borello et al. (136) demonstrate that near-UV–Visible absorption spectroscopy, combined with spectral deconvolution to quantify individual pigments (chlorophyll a/b, pheophytins, *β*-carotene, lutein), is a sensitive, rapid, non-destructive method to follow the evolution of pigment composition in EVOO from freshly pressed to on-the-shelf storage conditions. Over time, chlorophylls progressively degrade and convert into pheophytins, while carotenoids (e.g., β-carotene, lutein) show distinct degradation kinetics; these spectral changes correlate with storage time under realistic conditions (temperature, light, etc.). While conventional chemical analyses typically monitor oxidation or hydrolysis products, UV–Vis pigment analysis addresses a different dimension of oil quality deterioration the loss or transformation of naturally occurring pigments, which influences both the nutritional and sensory properties (color, minor compounds) of EVOO. The authors argue that UV–Vis spectroscopy thus provides an overview of oil aging during shelf-life, capturing pigment degradation that often precedes or accompanies oxidative deterioration.

#### 3.4.5 Innovative approach based on emerging spectroscopic techniques

Although conventional spectroscopic techniques have long been recognized as valuable alternatives to traditional analytical methods for edible oil assessment, recent technological advances have given rise to a new generation of spectroscopic tools that overcome previous analytical constraints and provide deeper insights into oil composition and quality. These novel approaches move beyond basic compositional profiling, enabling the precise detection of adulterants, contaminants and subtle molecular modifications that were previously difficult to identify using standard methodologies. The integration of state-of-the-art detection systems and innovative measurement strategies has markedly improved both sensitivity and specificity within complex oil matrices, thereby establishing more robust protocols for quality evaluation (137). Among the most notable developments is the application of different regions of the electromagnetic spectrum, particularly terahertz (THz) radiation and advanced fluorescence techniques. THz spectroscopy, which operates in the intermediate range between infrared and microwave frequencies (0.1–10 THz), provides unique molecular information by probing low-frequency vibrational modes and hydrogen-bonding interactions in oils. While earlier THz systems required bulky and stationary instruments. Innovations such as multiband metamaterial absorbers and hollow-core photonic crystal fiber sensors have led to compact and portable devices, allowing non-invasive, real-time monitoring of oil quality (138). In parallel, FS has advanced from large laboratory-based setups to compact sensors equipped with high-performance detectors such as charge-coupled devices and single-photon counting modules. These improvements have transformed fluorescence into a powerful analytical platform capable of generating three-dimensional spectral fingerprints through methods like Front-Face Fluorescence Spectroscopy and three-dimensional excitation–emission matrices (113). A further transformative step has been the convergence of spectroscopy with imaging technologies, particularly hyperspectral imaging (HSI). Unlike traditional spectroscopy, which yields bulk average measurement HSI combines spatial and spectral data to produce detailed chemical distribution maps of oil samples. Recent advancements have introduced compact and lightweight HSI systems equipped with prism-grating-prism (PGP) spectrometers and advanced detectors. These innovations have greatly improved portability, acquisition speed and applicability for real-time, on-site evaluations (139). Collectively, these emerging techniques share technological advancements that have strengthened their applicability in both laboratory and industrial contexts. The shift toward portable, field-deployable instruments has been particularly impactful, enabling rapid quality assurance while minimizing dependence on centralized laboratories. Furthermore, the use of advanced detector technologies and microfluidic integration has enhanced sensitivity, lowered detection thresholds, reduced sample volumes and increased throughput (115). As these approaches continue to evolve and become more widely accessible, they hold the potential to redefine quality monitoring in the edible oil sector by offering unprecedented analytical capabilities across production, distribution and storage stages (137). Chemometric and MVA have become indispensable tools in the characterization of edible oils, enabling accurate evaluation of quality, authenticity, and safety (140, 141). If combined with spectroscopic methods, these approaches facilitate the management and interpretation of complex datasets and support the development of robust predictive models. Core stages of MVA, such as data preprocessing, variable selection, model construction and performance validation, are increasingly strengthened by the incorporation of ML and DL algorithms. Preprocessing represents the initial and essential step in any chemometric workflow, as it ensures data consistency, reduces experimental noise, and minimizes variability in raw spectroscopic signals. Dimensionality reduction techniques such as Principal Component Analysis (PCA) are frequently applied to capture major sources of variation and focus subsequent modeling on the most informative features (85). Additional preprocessing methods, including Standard Normal Variate (SNV) and Multiplicative Scatter Correction (MSC), help mitigate scattering artifacts caused by physical differences among oil samples. After preprocessing, variable selection becomes a key step for improving efficiency and predictive power by eliminating irrelevant or redundant features. From literature, emerges that approaches such as the Successive Projection Algorithm (SPA) and Competitive Adaptive Reweighted Sampling (CARS) are commonly used in edible oil analysis. SPA minimizes collinearity among predictors, while CARS identifies the most discriminant variables through adaptive sampling. Iterative procedures like Iteratively Variable Subset Optimization (IVSO) and Bootstrapping Soft Shrinkage (BOSS) further refine feature selection, improving both the accuracy and interpretability of predictive models (85). Modeling constitutes the final stage of chemometric analysis, with ML and DL methods providing powerful tools for both qualitative (classification, discrimination) and quantitative (regression) applications. Support Vector Machines (SVM) and Convolutional Neural Networks (CNN) are particularly effective for classification, whereas PLS regression and Deep Neural Networks (DNN) demonstrate strong performance in quantitative predictions. PCA remains widely employed for exploratory analysis and pattern recognition tasks. Moreover, data fusion strategies that integrate information from multiple spectroscopic platforms, such as NIR, Raman and FT-IR, further strengthen predictive accuracy and broaden analytical scope. Rigorous validation of these models ensures their applicability in real-world scenarios, ultimately supporting reliable monitoring of edible oil quality, safety and authenticity (85). The integration of multiple non-destructive techniques (e.g., NMR, E-nose, Fluorescence) in combination with chemometric models and the use of ML algorithms is opening new possibilities for reliable predictive models of PSL and SSL. These approaches allow for a more sustainable, rapid, and real-time assessment, reducing the need for classical chemical analysis. Their potential is particularly interesting for the oil industry, where efficiency and sustainability are increasingly central (142).

#### 3.4.6 Raman spectroscopy

RS represents a complementary approach to infrared spectroscopy for evaluating the safety and quality of edible oils. The technique is based on the inelastic scattering of monochromatic light, generating Raman spectra that serve as molecular fingerprints of oil composition and molecular interactions. The resulting spectrum contains characteristic bands corresponding to specific molecular vibrations, thereby providing specific insights into the structural and compositional features of the sample (85). RS discriminates among fatty acids through their specific vibrational fingerprints, in particular monounsaturated (MUFAs) and polyunsaturated fatty acids (PUFAs) present distinctive spectral features linked to double bonds, whereas saturated fatty acids (SFAs) display signatures characteristic of their fully saturated structures (143). Recent advances in Raman instrumentation, including the development of more powerful laser sources, enhanced fiber-optic probes and highly sensitive detectors, have meaningfully improved the signal-to-noise ratio for edible oil analysis (85). Through this mechanism, RS enables the precise identification of molecular structures and compositions, where its application in edible oil quality and safety assessment is highlighted (85). Eggertson and Venturini (144) conducted a study on how Resonant Raman Spectroscopy (RRS) is useful for monitoring changes in carotenoids during the accelerated aging of EVOO. Raman spectra were acquired under resonance conditions (excitation at 488 nm) to amplify the typically weak signal of carotenoids. Signal analysis allowed the isolation of Raman peaks from the fluorescent background and the evaluation of the evolution of carotenoids and fatty acids during storage. This study conducted by Eggertson and Venturini (144) demonstrated that RRS is a promising technique for the rapid, sustainable, and non-destructive evaluation of the shelf life of EVOO, reducing dependence on destructive and solvent-intensive methods such as HPLC.

#### 3.4.7 Electronic nose

Given the limitations of previously reported techniques, there is an increasing demand for new methodologies that are non-destructive, low-cost, rapid, and broadly accessible, while also being suitable for commercial applications. In this context, electronic sensing technologies, including E-Nose, tongues and eyes, have been widely explored for applications in quality control, process monitoring, SL prediction and authenticity verification (145). The E-Nose, designed to emulate human olfaction, employs non-selective or semi-selective sensor arrays that generate electronic signals in response to volatile compounds (146). Various studies have applied E-Nose technology to assess oxidation or SL in a wide range of products, including canola oil, EVOO, vinegar, wine (145). This study investigates the use of E-Nose technology combined with multivariate analyses, including linear discriminant analysis (LDA), quadratic discriminant analysis (QDA), and support vector machine (SVM) to predict edible oil SL. The obtained results were validated against the standards of the American Oil Chemists Society (AOCS). The proposed methodology demonstrates potential as non-destructive alternative for monitoring the quality and SL of oils throughout the storage and distribution chain (145). Several studies have demonstrated the ability of the E-nose to discriminate between fresh and oxidized oils, correlating sensor signals with traditional chemical parameters and sensory perception (147). The E-nose is a technology that, when properly trained, allows oils to be classified according to their state of oxidation by recognizing odors. The volatile phase produced by a food contains a wealth of information about its composition and the physical, chemical, and biological processes affecting it. The E-nose, as its name suggests, is a tool whose operating principle is inspired by the sense of smell. It consists of two main components: a matrix of sensors and a pattern recognition algorithm, which perform the functions of the receptors present in the olfactory epithelium and the processing performed by the brain, respectively. The sensors in the array must be partially selective and different from each other, i.e., each with its own partial selectivity different from that of the other sensors. Thanks to this characteristic, each odor is encoded by the sensors according to a specific distribution of responses, called the olfactory fingerprint of the odor. Each smell has its own olfactory fingerprint and thanks to this uniqueness, pattern recognition software is able to discriminate between different smells. The E-nose, like our sense of smell, is a tool that learns from experience. The development of this instrumentation is carried out according to a training and validation process (148). During the training phase, the device is exposed to the characteristic odors of the application of interest, in order to build a dataset of reference olfactory fingerprints. Pattern recognition algorithms are used to construct a mathematical model that allows the device to identify the correspondences between each olfactory fingerprint and the corresponding odor. In the subsequent validation phase, the developer exposes the nose to different odors and verifies the instrument is ability to recognize them correctly. At that point, the instrument is ready for use and can work autonomously, without the need for specialized personnel. The training phase is fundamental for this instrumentation: the nose must learn to recognize target odors even in the presence of interferents or anomalous situations, which could otherwise lead to incorrect classifications. The scientific literature on E-nose reports many case studies demonstrating the potential of this technology, for example to assess the correct degree of ripeness of coffee (149) but also to recognize the possible adulteration of EVOO through the addition of less valuable oils (150). It should be emphasized that the E-nose is not intended to replace analytical techniques such as gas chromatography. It does not have the same precision and reliability as these techniques but, once trained, it can be used, even in the field, as a screening technique that is faster and cheaper than analytical techniques, providing an immediate response in a shorter time, thus leaving only the most complex cases to be investigated in the laboratory (150).

## 4 Conclusion and future perspectives

In recent years, non-destructive analytical techniques have demonstrated growing potential in ensuring the quality and authenticity of extra virgin olive oil (EVOO), addressing the limitations of traditional methods, which are destructive, risky for operators and the environment, time-consuming, and incompatible with real-time monitoring. Spectroscopic techniques such as NIR, MIR FT-IR, Raman, fluorescence, NMR, and electronic sensors have shown remarkable progress in terms of speed, sustainability, and the ability to generate many multidimensional information on the composition and oxidative dynamics of oils. Integration with chemometrics and artificial intelligence algorithms (machine learning, deep learning, etc.) has further enhanced the accuracy of the results, making it possible to develop more robust models for classification, authentication, and shelf life prediction. Despite progress, there are still some difficulties to overcome. These non-destructive techniques can be influenced by cross-interfering factors such as variety, container, or storage environment, which alter the results and make measurements less reliable, the so-called “matrix effect.” In addition, accurate data requires robust calibration models built on a large and diverse number of samples. Another challenge is the lack of standardized protocols: different instruments and different methods of analysis can lead to results that are not always comparable. Finally, these new techniques should be compared on a large scale with officially recognized methods (such as those established by the EU or the Codex Alimentarius). Only by demonstrating that they are equivalent or better compared to current standards can they be accepted by the authorities and become an integral part of official and industrial controls. Current research highlights the growing importance of non-destructive technologies and their developments, which represent a promising direction, especially for industrial applications and real-time quality monitoring. Future research should also focus on addressing the lack of predictive models for secondary shelf life (SSL), integrating both environmental factors and the influence of packaging. In this regard, the development of predictive models capable of assessing both primary and secondary shelf life, taking into account the interaction with smart and active packaging systems, could represent an innovative approach. Furthermore, the integration of these analytical technologies with smart, active, and sustainable packaging solutions could enable a quality monitoring and traceability system on an industrial scale.
